# 55-year-old Male with Exertional Dyspnea

**DOI:** 10.5811/cpcem.2020.3.46393

**Published:** 2020-04-23

**Authors:** Eric R. Friedman, J. David Gatz, Zachary D.W. Dezman, Laura J. Bontempo

**Affiliations:** * University of Maryland Medical Center, Department of Emergency Medicine, Baltimore, Maryland; † University of Maryland School of Medicine, Department of Emergency Medicine, Baltimore, Maryland

**Keywords:** Clinicopathological cases, cardiology, epidemiology

## Abstract

**Introduction:**

Dyspnea is a common presenting complaint for many patients in the emergency department.

**Case Presentation:**

A 55-year-old man with type I diabetes presented to the emergency department with one month of intermittent palpitations and dyspnea. His lungs were clear to auscultation, and his chest radiograph was normal.

**Discussion:**

This case takes the reader through the differential diagnosis and systematic work-up of dyspnea with discussion of the diagnostic study, which ultimately led to this patient’s diagnosis and successful treatment.

## CASE PRESENTATION (Dr. Eric R. Friedman)

A 55-year-old man presented to the emergency department (ED) of an urban, academic, medical center with intermittent palpitations (fast beats accompanied by a “pounding” sensation in the chest) for the prior month. Palpitations were most noticeable at night but had become more severe and constant over the preceding three days. He also reported shortness of breath while supine in bed and lightheadedness with exertion. The patient is an avid hiker, so he only presented once his dyspnea prevented him from completing a typical family hike. He denied any chest pain, fevers, weight loss, syncope, leg swelling, cough, nausea, vomiting, or rashes.

The patient was diagnosed with type 1 diabetes mellitus when he was 30 and has a history of hypertension. He was taking insulin glargine nightly with sliding-scale insulin aspart during meals and had recently switched from ramipril to losartan 50 milligrams (mg) daily due to nocturnal cough. He denied any drug, alcohol, or tobacco use. His father died suddenly at age 42 due to a heart attack. He denied any allergies.

The patient’s vital signs on presentation were as follows: temperature 97.2 degrees Fahrenheit; blood pressure 131/83 millimeters of mercury (mm Hg); pulse 55 beats per minute, respiratory rate 18 breaths per minute; and oxygen saturation 99% on room air, with a body mass index of 28.6 kilograms per meters squared. Physical exam revealed a well-developed and well-nourished male patient in no acute distress. His head was normocephalic and atraumatic. His eye exam was normal with pupils that were equal, round, and reactive to light. No scleral icterus was seen. His neck was supple and had normal range of motion. There was no jugular venous distension seen. The patient’s cardiac exam was notable for a bradycardic and irregularly irregular heartbeat without a murmur. His lungs were clear to auscultation bilaterally, and no wheezes or rales were heard. The patient’s abdomen was soft, non-tender, and non-distended. His extremities were warm and well perfused, and without edema. The patient’s cranial nerves II–XII were intact, and gait and strength assessments were unremarkable. No pronator drift was seen. Skin was normal in appearance, without any lesions or rashes. The patient demonstrated a normal mood and affect throughout the history and exam.

**Image 1:**
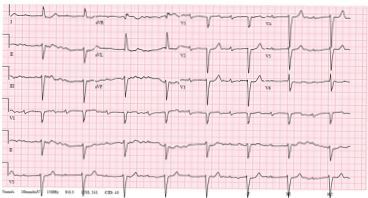
Electrocardiogram of a 55-year-old male with palpitations and dyspnea, taken while in the emergency department.

**Image 2:**
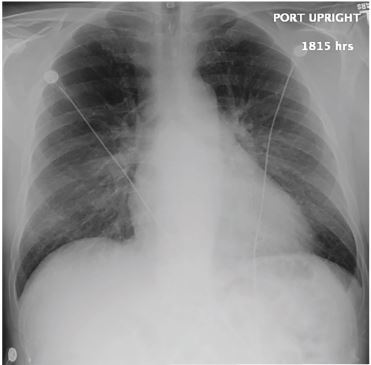
Anterior-posterior chest radiograph of a 55-year-old male with palpitations and dyspnea.

**Image 3:**
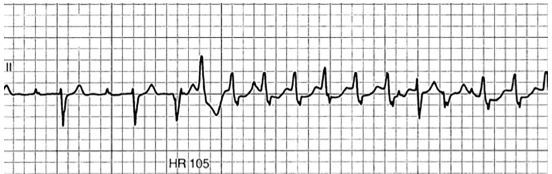
Patient’s cardiac rhythm during an episode of palpitations that occurred while he was in the emergency department. ”HR 105” refers to the patient’s heart rate.

A chest radiograph (CXR) and electrocardiogram (ECG) were obtained (Images 1 and 2, respectively). Laboratory studies (Table) were notable for a white blood cell count of 6.2 thousand per microliter, hemoglobin of 15.1 grams per deciliter, platelets of 190,000 per microliter, glucose 250 milligrams per deciliter, negative troponin, B-type natriuretic peptide (BNP) of 502 picograms per deciliter, a thyroid stimulating hormone level of 1.2 milli-international units per liter with free thyroxine of 1.4 nanomoles per liter, and a *Borrelia Burgdorferi* IgM/IgG (immunoglobulin) titer of 0.28, negative antinuclear antibody test. (The latter test resulted after the patient was admitted.) While in the ED the patient experienced an acute episode of palpitations. His rhythm, as recorded by telemetry, is shown in Image 3. An echocardiograph was performed, which showed severe global hypokinesis with a left ventricular ejection fraction (LVEF) of 15%, mild dilation, biatrial enlargement, and no pericardial effusion.

## FACULTY DISCUSSION (Dr. J. David Gatz)

I can feel my own heart racing as I work through this case. A passionate hiker myself, I immediately empathize with this patient’s concern of diminishing trail endurance. It is all quite alarming for a relatively young gentleman who otherwise seems healthy and active. He goes outdoors frequently and only has two chronic medical problems – insulin-dependent diabetes and hypertension. He did have a recent medication change, from ramipril to losartan, but this seems unlikely to be significant. It is alarming that his father suffered a cardiac-related death at such a young age (only 42 years old!) and raises the question of a potential hereditary component to this presentation. His reported symptoms unfortunately don’t give us much additional direction: A month of palpitations with some exertional pre-syncope and orthopnea are rather vague. On exam, the patient’s vital signs are frustratingly benign. The respiratory rate of 18 breaths per minute is probably just an estimated number and not directly measured. He is slightly bradycardic at 55 beats per minute, which may be due to his baseline level of athletic activity. He is slightly hypertensive, but this is a known diagnosis. His physical exam appears normal except for an irregular rhythm on cardiac assessment. His labs are mostly benign. Mild hyperglycemia is reasonable in the setting of his diabetes. His Lyme titers are within normal ranges. The patient’s BNP, while not drastically elevated, is certainly higher than what we would expect in an otherwise healthy individual without an existing cardiac or renal diagnosis.

The patient’s ECG is abnormal and difficult to interpret. At first glance it appears to show a sinus bradycardia with first-degree atrioventricular (AV) block. There is left ventricular hypertrophy with repolarization abnormalities, and a left anterior fascicular block. Then, as if by premonition, the patient experiences a ventricular tachycardia. Understandably this triggers additional work-up including a CXR and an echocardiogram.

This is a large amount of information to work through, and it becomes essential to not miss the forest for the trees. Combining the patient’s initially vague symptoms with the left ventricular hypertrophy on his ECG, elevated BNP, and dilated hypokinetic echo, it is clear that this patient has a cardiomyopathy. The question becomes why? Something has made his cardiac tissue structurally and functionally abnormal, yet in the absence of any known coronary artery disease, valvular disease, or hypertension (currently just on monotherapy with only mildly elevated pressures in the ED). Cardiomyopathy is typically thought of as falling within one of three general categories: hypertrophic, dilated, or restrictive.^1^ The patient’s ECG does not demonstrate the classic findings we would expect in a septal or apical variant of hypertrophic cardiomyopathy.^2^ While there is an AV block and widened QRS complexes, the patient’s ECG lacks the small voltages typically associated with a restrictive cardiomyopathy. Given that the patient’s echo demonstrates global hypokinesis (not apical) and there are no known significant new stressors in his life, his presentation does not seem consistent with a stress (Takotsubo) cardiomyopathy. Given all of this, it seems reasonable to narrow our search to potential causes of dilated cardiomyopathy (DCM).

But the number of etiologies just within DCM is long.^3^ There are many known infectious causes (bacterial, viral, spirochetal, rickettsial, mycotic, protozoal, helminthic), but the patient has no fevers, antecedent illnesses, or laboratory evidence of infection. Lyme disease is an exciting thought given the patient’s outdoor activities, but the patient’s Lyme titers are within normal limits. While deposition diseases (hemochromatosis/amyloidosis) can cause DCM, there is no ECG evidence or associated stigmata. Numerous medications (especially chemotherapeutics and antiretrovirals) are known to cause DCM, but the patient is not taking any of them. Similarly, the patient has no history to suggest ingestion or exposure to toxins that could potentially cause DCM. Laboratory results and history exclude DCM secondary to any profound electrolyte, renal, or nutritional abnormalities. Diabetes can cause DCM, and while we don’t have a hemoglobin A1c to show whether his diabetes is controlled or not, it would be unlikely for a patient to present with DCM without a history of coronary artery disease, myocardial infarction, or any of the more common complications of diabetes (e.g., peripheral neuropathy, diabetic nephropathy, and retinopathy). There is no evidence of additional endocrine or genetic disorder. This leaves one final major category of potential etiologies – autoimmune processes.

The autoimmune diseases that cause DCM include systemic lupus erythematosus (SLE), dermatomyositis, scleroderma, rheumatoid arthritis, and sarcoidosis. SLE is less likely because the patient doesn’t have the classic symptoms, the rash, or nephritis. Similarly, the classic cutaneous findings of dermatomyositis and scleroderma are not present. Sarcoidosis, however, can be much more subtle. With this in mind, the patient’s CXR seems to provide an essential clue.

Hilar structures on CXRs can be confusing. This patient’s CXR demonstrates additional “lumpy and bumpy” radiodensities in the hilar areas. In contrast to the smooth contours of bilateral pulmonary artery enlargement, these findings are consistent with hilar adenopathy, a finding suggestive of a neoplastic, infectious (tuberculosis, histoplasmosis), and inflammatory processes like sarcoidosis.^4^

And thus, while this has been a long and winding path through the woods, we appear to have finally stumbled into a clearing. Sarcoidosis uniquely unifies our clinical suspicion of a DCM with the patient’s CXR. Moreover, the patient is the appropriate age and has experienced many of the typical symptoms including heart failure, AV blocks, and arrythmias. Confirming the diagnosis can be attempted by cardiac biopsy, the classic diagnostic test, but this seems excessively invasive. Cardiac magnetic resonance imaging (MRI), however, has reasonable accuracy and is therefore my preferred diagnostic test. Hopefully a correct diagnosis and treatment will let this patient return to the trail for, as astutely noted by Hippocrates, “Walking is man’s best medicine.”

## CASE OUTCOME (Dr. Friedman)

The patient was admitted to the cardiology service. Cardiac catherization was considered, but the patient instead received a cardiac MRI that showed DCM with extensive enhancement involving inferoseptal, inferior, and inferolateral segments from base to apex and mediastinal lymphadenopathy, with a severely decreased ventricular function and a LVEF of 21%. The patient had multiple runs of ventricular tachycardia while admitted and consequently received an automated implantable cardioverter-defibrillator (AICD). A hilar lymph node biopsy showed non-caseating granulomatous inflammation and multinucleated giant cells, consistent with sarcoidosis. The patient was originally started on prednisone 40 milligrams (mg) for the sarcoidosis and alprazolam 1 mg as needed due to post-traumatic stress disorder from his recurrent AICD shocks. He has since transitioned to 1 gram of mycophenolate mofetil and prednisone 35 mg daily. He is doing well and has not experienced any recent shocks from his AICD.

## RESIDENT DISCUSSION

Sarcoidosis has a protean presentation, and the incidence of the disease varies widely among different cultural and racial groups: 1–2 persons per 100,000 of Japanese descent are affected, while 35–80 per 100,000 African-American persons are affected.^5^ Cardiac sarcoidosis is relatively rare, occurring in less than 5% of all cases.^5^ Untreated cardiac sarcoidosis can result in sudden cardiac death from ventricular tachyarrhythmias or atrioventricular blocks, and should be considered in patients with unexplained low LVEF, unexplained sustained ventricular tachycardia, and new AV block (usually Mobitz type II second degree or third degree).^6^ Endomyocardial biopsy has the highest specificity for the diagnosis of cardiac sarcoidosis but is rarely performed given the comparative ease and lack of complications associated with cardiac MRI.^7^ Cardiac MRI has an acceptably high sensitivity and specificity of 89% and 78%, respectively.^7^ Cardiac MRI will usually show multiple areas of enhancement in a non-infarct pattern and will show direct enhancement from the left ventricle, across the interventricular segment, into the right ventricle.^8^

Early treatment with immunosuppressants such as steroids can help treat cardiac sarcoidosis. In one case, a 32-year-old man with cardiac sarcoidosis had complete resolution of his AV block and ventricular tachycardia within two weeks of initiating steroid treatment. Follow-up cardiac MRI showed no further uptake, consistent with resolution of his cardiac sarcoidosis.^9^ This case illustrates a rare, deadly disease that was fortunately recognized early. The correct treatment was started, which resolved his symptoms and dysrhythmias.

## FINAL DIAGNOSIS

Cardiac sarcoidosis.

## KEY TEACHING POINTS

1. Consider cardiac sarcoidosis in younger patients presenting with symptoms of heart failure, arrhythmias, or syncope.
2. Up to a quarter of cardiac sarcoidosis cases may occur in the absence of any extracardiac involvement.
3. The most typical radiographic features of thoracic sarcoidosis include symmetric hilar and mediastinal lymphadenopathy.

## Figures and Tables

**Table:** Laboratory results of a 55 year-old male with palpitations and dyspnea.

Lab test	Value	Units	Normal range
White blood cell count	6.2	K/mcL	4.5 – 11.0
Hemoglobin	15.1	g/dL	12.6 – 17.4
Hematocrit	43.7	%	37.0 – 50.0
Mean corpuscular volume	85.7	fL	80.0 – 96.0
Mean corpuscular hemoglobin	29.6	pg	28.0 – 33.0
Mean corpuscular hemoglobin concentration	34.6	g/dL	33.0 – 36.0
Platelets	190	K/mcL	153 – 367
Mean platelet volume	11.6	fL	9.4 – 12.4
Red cell distribution width	13.3	%	12.0 – 15.2
Sodium	141	mmol/L	136 – 145
Potassium	4.5	mmol/L	3.5 – 5.1
Chloride	105	mmol/L	98 – 107
Bicarbonate	27	mmol/L	21 – 30
Glucose	250	mg/dL	70 – 99
Creatinine	1.01	mg/dL	0.66 – 1.25
Blood urea nitrogen	16	mg/dL	7–20
Calcium	9.2	mg/dL	8.6 – 10.2
Total protein	6.1	g/dL	6.3 – 8.2
Albumin	3.4	g/dL	3.5 – 5.2
Bilirubin total	0.9	mg/dL	0.3 – 1.2
Alkaline phosphatase	63	units/L	38 – 126
Aspartate aminotransferase	25	units/L	17 – 59
Alanine aminotransferase	40	units/L	21 – 72
Thyroid stimulating hormone	1.2	mIU/L	0.47 – 4.68
T4 free	1.4	ng/dL	0.6 – 2.5
Magnesium	1.8	mg/dL	1.6 – 2.6
Phosphorus	4.2	mg/dL	2.5 – 4.5
*Borrelia burgdorferi* IgG/IgM Total*	0.28		
Anti-nuclear antibody	Neg		
Brain natriuretic peptide	502	pg/mL	<900
Troponin	<0.02	ng/mL	<0.07

* The amount of immunoglobulin G or M that are reactive to Borrelia antigen, results < 0.9 of the laboratory standard are considered negative.

*K*, thousand; *mcL*, microliter; *g*, grams; *dL*, deciliter; *fL*, femtoliter; *pg*: picogram; *mmol*, millimoles; *L*, liter; *mg*, milligrams; *mIU*, milli-international unit; *ng*, nanograms; *pg*, picograms; *mL*, milliliter; *ng*, nanograms; *Ig*: immunoglobulin; *Neg*, nagative.
